# Integrating Genomics and Biogeography to Unravel the Origin of a Mountain Biota: The Case of a Reptile Endemicity Hotspot in Arabia

**DOI:** 10.1093/sysbio/syae032

**Published:** 2024-07-02

**Authors:** Bernat Burriel-Carranza, Héctor Tejero-Cicuéndez, Albert Carné, Gabriel Mochales-Riaño, Adrián Talavera, Saleh Al Saadi, Johannes Els, Jiří Šmíd, Karin Tamar, Pedro Tarroso, Salvador Carranza

**Affiliations:** Institute of Evolutionary Biology (CSIC-Universitat Pompeu Fabra), Passeig Marítim de la Barceloneta, 37–49, 08003, Barcelona, Spain; Museu de Ciències Naturals de Barcelona, P° Picasso s/n, Parc Ciutadella, 08003, Barcelona, Spain; Institute of Evolutionary Biology (CSIC-Universitat Pompeu Fabra), Passeig Marítim de la Barceloneta, 37–49, 08003, Barcelona, Spain; Department of Biodiversity, Ecology and Evolution, Faculty of Biology, Universidad Complutense de Madrid, 28040 Madrid, Spain; Museo Nacional de Ciencias Naturales (MNCN), CSIC, C/ José Gutiérrez Abascal 2, 28006, Madrid, Spain; Institute of Evolutionary Biology (CSIC-Universitat Pompeu Fabra), Passeig Marítim de la Barceloneta, 37–49, 08003, Barcelona, Spain; Institute of Evolutionary Biology (CSIC-Universitat Pompeu Fabra), Passeig Marítim de la Barceloneta, 37–49, 08003, Barcelona, Spain; Environment Authority, Muscat, Oman; Breeding Centre for Endangered Arabian Wildlife, Environment and Protected Areas Authority, Sharjah, United Arab Emirates; Department of Zoology, Faculty of Science, Charles University, 128 00, Prague, Czech Republic; Institute of Evolutionary Biology (CSIC-Universitat Pompeu Fabra), Passeig Marítim de la Barceloneta, 37–49, 08003, Barcelona, Spain; CIBIO,Centro de Investigação em Biodiversidade e Recursos Genéticos,InBIO Laboratório Associado, Campus de Vairão, Universidade do Porto, 4485-661 Vairão, Portugal; BIOPOLIS Program in Genomics, Biodiversity and Land Planning, CIBIO, Campus deVairão, 4485-661 Vairão, Portugal; Institute of Evolutionary Biology (CSIC-Universitat Pompeu Fabra), Passeig Marítim de la Barceloneta, 37–49, 08003, Barcelona, Spain

**Keywords:** Arabia, arid environments, biogeography, ddRADseq, desertification, genomics, mountain building, reptiles, Hajar Mountains

## Abstract

Advances in genomics have greatly enhanced our understanding of mountain biodiversity, providing new insights into the complex and dynamic mechanisms that drive the formation of mountain biotas. These span from broad biogeographic patterns to population dynamics and adaptations to these environments. However, significant challenges remain in integrating large-scale and fine-scale findings to develop a comprehensive understanding of mountain biodiversity. One significant challenge is the lack of genomic data, especially in historically understudied arid regions where reptiles are a particularly diverse vertebrate group. In the present study, we assembled a de novo genome-wide SNP dataset for the complete endemic reptile fauna of a mountain range (19 described species with more than 600 specimens sequenced), and integrated state-of-the-art biogeographic analyses at the population, species, and community level. Thus, we provide a holistic integration of how a whole endemic reptile community has originated, diversified and dispersed through a mountain system. Our results show that reptiles independently colonized the Hajar Mountains of southeastern Arabia 11 times. After colonization, species delimitation methods suggest high levels of within-mountain diversification, supporting up to 49 deep lineages. This diversity is strongly structured following local topography, with the highest peaks acting as a broad barrier to gene flow among the entire community. Interestingly, orogenic events do not seem key drivers of the biogeographic history of reptiles in this system. Instead, past climatic events seem to have had a major role in this community assemblage. We observe an increase of vicariant events from Late Pliocene onwards, coinciding with an unstable climatic period of rapid shifts between hyper-arid and semiarid conditions that led to the ongoing desertification of Arabia. We conclude that paleoclimate, and particularly extreme aridification, acted as a main driver of diversification in arid mountain systems which is tangled with the generation of highly adapted endemicity. Overall, our study does not only provide a valuable contribution to understanding the evolution of mountain biodiversity, but also offers a flexible and scalable approach that can be reproduced into any taxonomic group and at any discrete environment.

## Introduction

The patterns of life on Earth are intrinsically connected to its landscape features and, among those, mountains rise as especially important components (Rahbek et al. 2019a). Mountains are inherently unstable systems, constantly changing due to tectonic, erosional, and climatic processes under short geological periods of time. This results in highly heterogeneous and transient environments that can repeatedly lead to the split and isolation of species ranges, to evolutionary adaptation to changing environments and, consequently, to diversification (Perrigo et al. 2020). Not surprisingly, mountains are home to staggering levels of species richness, containing about 85% of the global vertebrate diversity (Rahbek et al. 2019a).

While mountains are undoubtedly a cornerstone to understand the origin and evolution of biodiversity and, particularly, the emergence of endemicity (Korner and Spehn 2002; Antonelli 2015; Favre et al. 2015; Noroozi et al. 2018; Rahbek et al. 2019b), our understanding on how these species-rich systems originate still remains limited (McCain 2010; Rahbek et al. 2019b; Perrigo et al. 2020). On the one hand, several studies show that the buildup of biodiversity correlates with the uplift history of the mountain range. In these cases, mountain diversity is postulated to be either the result of dispersal of pre-adapted montane species to newly formed habitats with subsequent speciation (Merckx et al. 2015; Roycroft et al. 2022), or the result of passive uplift of lowland populations adapting to new environments at the same pace as the mountain rises (Ribas et al. 2007; Heads 2019). On the other hand, some studies show that many montane species radiations post-date the formation of their physical landscape and suggest that past climatic conditions have had a major role in the assembly of those communities (Madriñán et al. 2013; Hughes and Atchison 2015). Altogether, both geological and paleoclimatic events seem to play important roles on the evolution and distribution of biodiversity in these systems. Therefore, in order to fully grasp the complex and dynamic processes leading to the accumulation of mountain biodiversity, a variety of interdisciplinary approaches ranging from evolutionary insight to paleoclimate and geological views are required.

However, several challenges usually related to the intrinsic complexity of mountain systems still remain. For instance, the complex topography of most mountain ranges as well as the high levels of small-ranged endemic species (micro-endemics), tend to difficult sampling, often requiring arduous and long fieldwork (Carranza et al. 2018). Not surprisingly then, studies that encompass entire guilds or biotas are not abundant (e.g., Merckx et al. 2015; Ding et al. 2020; Li et al. 2021). Another challenge is that studies of mountain diversity tend to be biased towards hyperdiverse tropical mountains (Merckx et al. 2015; Roycroft et al. 2022) or emblematic mountain ranges (Esquerré et al. 2019). In contrast, mountains in arid regions of North Africa and Arabia have been historically overlooked (McCain 2010; Brito et al. 2014). Such disparity shortsights our understanding of mountain diversity as it has been shown that the extreme climatic conditions of arid mountains seem to promote distinct biodiversity patterns across the elevational gradient than those observed in more humid mountains, especially in the case of reptiles (McCain 2010; Tallowin et al. 2017; McDonald 2021). Therefore, further research on arid mountain ranges can improve our understanding of global overarching processes influencing the buildup of mountain biodiversity.

Advances in genomics can substantially improve our understanding of the buildup of montane communities. The number of SNPs that can be recovered through Next Generation Sequencing (NGS) techniques greatly surpasses those obtained through traditional Sanger sequencing, providing unparalleled resolution to disentangle phylogenetic relationships, detect mito-nuclear discordances, uncover cryptic diversity, or retrieve fine-scaled resolution of population structure, among other benefits (Allendorf et al. 2010; Stöck et al. 2012; Chattopadhyay et al. 2016; Vilaça et al. 2021). However, significant challenges remain in integrating large-scale genomic data together with the biogeographic insights needed to understand the processes of colonization, dispersal and diversification of montane communities (Perrigo et al. 2020). For instance, although the availability of reference genomes and whole genome sequencing (WGS) data is exponentially increasing (Gable et al. 2023), there is still not enough available data to produce well represented phylogenies from highly divergent taxa (e.g., a phylogeny for all squamates), thus hindering the implementation of biogeographic studies over entire guilds or biotas. The cost of producing WGS data can also be a drawback for studies that rely on extensive sampling (Card et al. 2023). Genomic reduced representations (such as restriction-site-associated DNA sequencing) offer a cost-effective solution to include dozens to hundreds of specimens in genomic studies and have become popular among researchers, especially when working with non-model organisms (Miller et al. 2007; Davey et al. 2011). However, since these methods rely on targeting specific genomic regions given by the restriction enzymes used, as phylogenetic distance among the studied taxa increases, the number of homologous loci recovered decreases, greatly difficulting the reconstruction of deep phylogenies. Therefore, the use of genomics to study montane diversity seems to be limited to a small number of samples (Ericson and Irestedt 2022; Nannan et al. 2022), to a few closely related species (Mastretta-Yanes et al. 2018), or to species complexes stemming from relatively young diversification events (Roycroft et al. 2022).

Efforts to include the fine-scale resolution obtained through NGS into studies of mountain diversity could benefit from the integration of both Sanger sequencing and genomic reduced representations. Synergistic analyses using first Sanger sequencing data to reconstruct deep-time phylogenies can provide a robust framework to determine the origin and timing of first colonization events. Then, subsequent dispersal, adaptation, and diversification can be investigated with thousands of SNPs retrieved from genomic sequencing targeting specific focal groups stemming from independent colonization events.

**Figure 1.**
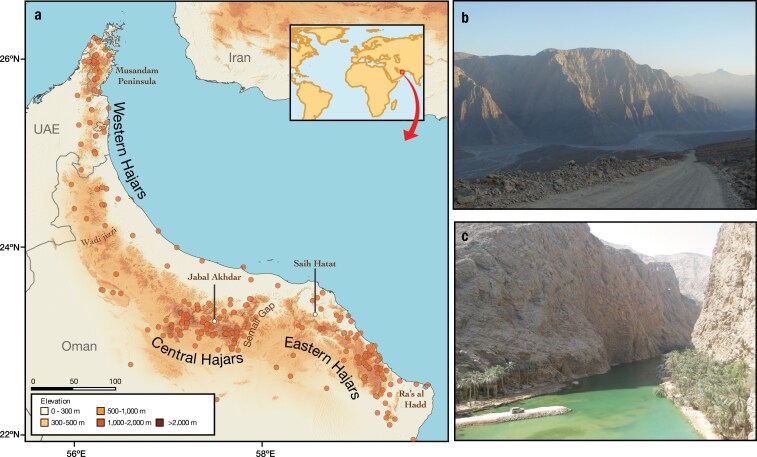
Area of study. a) Regional topographic map of the Hajar Mountains of southeastern Arabia showing the main topographic features in the region. Points represent sampled localities, and the two highest peaks are highlighted with white circles. Inset map shows the location of the Hajar Mountains in a global context. b) Picture of the Hajar Mountains showing the arid and steep landscape of its topographic features. The picture was taken in the Musandam Peninsula by J.S. c) Wadi Ash Shab in the Eastern Hajars (taken by S.C.). These deep canyons are mostly ephemeral and surface water only flows during periods of short, but intense rain.

Within the Arabian Peninsula, which is comprised of 99% arid or hyper-arid environments (Kotwicki and al Sulaimani 2009), mountain ranges likely act as important diversity reservoirs, as many species benefit from the somewhat milder climatic conditions provided by high altitudes in an otherwise torrid and dry environment. Among those, the Hajar Mountains of southeastern Arabia constitute an outstanding hotspot of endemicity in the peninsula (Šmíd, et al. 2021). Isolated by the widest continuous sand desert in the world (the Rub’ Al Khali), the Hajar Mountains conform an exceptionally arid mountain system spanning across the northern coast of Oman and the UAE (Fig. 1). This mountain range has an ancient and dynamic geological history linked to the Arabian-Eurasian plate collision, with the current topography of the mountain range originating about 40 million years ago (ma; Hansman et al. 2017) followed by several secondary uplifts during the Neogene (Bosworth et al. 2005; Glennie 2006; Jacobs et al. 2015; Hansman et al. 2017). However, an early proto-Hajar Mountains could have originated as early as 90 ma (Hansman et al. 2017).

The Hajar Mountains harbor high diversity in various plant and animal groups (Ghazanfar 1991; Gebauer et al. 2007; Brinkmann et al. 2009; Carranza et al. 2018, 2021; Burriel-Carranza et al. 2019, 2022), with reptiles standing out as the most diverse vertebrate group in the system with high levels of cryptic diversity and endemicity. Indeed, with up to 19 described endemic species, the Hajars constitute a hotspot of reptile endemicity in Arabia (Carranza et al. 2021; Šmíd et al. 2021; Burriel-Carranza et al. 2022) and in all the Western Palearctic (Ficetola et al. 2018).

Owing to their relatively small size, complex topography, and isolation from other mountains, the Hajar Mountains constitute the perfect laboratory to study the patterns and drivers of reptile diversity in arid mountains as well as the fine-scale processes that contribute to the buildup of mountain biodiversity. In this study, we dissect the evolutionary history and biogeography of the endemic reptile community of the Hajar Mountains through an integrative approach, merging both Sanger sequencing and genomic data. We infer a comprehensive phylogeny of squamate reptiles (order Squamata), and generate genome-wide ddRADseq data for all endemic reptile species (19 described species; more than 600 specimens sequenced) inhabiting the mountain range to (i) assess the population structure, phylogenomic relationships, diversity, and landscape genomics of its complete endemic reptile fauna; (ii) disentangle the biogeographic and evolutionary dynamics of this guild at different levels (population, species, and community); and (iii) reconstruct the biogeographic events of colonization, dispersal, and diversification that have led this mountain range into one of the most important hotspots of reptile diversity and endemicity in the Western Palearctic (Ficetola et al. 2018).

## Materials and Methods

### Study Design

**Figure 2.**
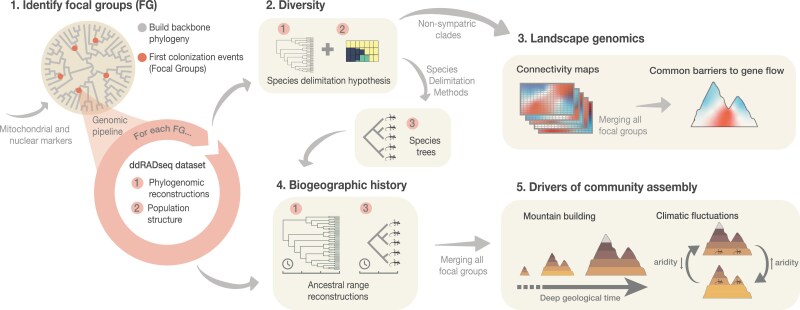
Graphical representation of the workflow conducted for the present study. We implemented a hierarchical approach to reveal the origin and evolution of a reptile community in a mountain range. First, nuclear and mitochondrial markers were used to infer a squamate backbone phylogeny, and focal groups (FG) were identified through an ancestral state reconstruction of mountain occupancy on the inferred phylogeny (Step 1). Then, genome-wide ddRADseq datasets were assembled to reveal the diversity, systematics and population structure of each FG (Step 2). Integrating genomic datasets, common barriers to geneflow were identified through landscape genomics (Step 3). Furthermore, the biogeographic histories of each FG were reconstructed (Step 4) and merged together to elucidate the biogeographic patterns of the entire reptile community. Finally, we interrogated the effects of mountain building and climate fluctuations as possible drivers of deep- and early-time community assembly respectively (Step 5).

This study follows an integrative approach that combines top-down and bottom-up strategies to unravel the evolutionary history and biogeography of the Hajar Mountains’ reptile community. Specifically, the study was conducted through the following steps (see Fig. 2 for a graphical representation):

1. Identification of focal groups: We reconstructed a time-calibrated backbone squamate phylogeny with mitochondrial and nuclear markers to determine the number of focal groups in the system. These were defined as species or lineages currently distributed within the Hajars originating from a common colonization event.
2. Genomic dataset assembly: Genome-wide ddRADseq datasets were assembled for each focal group, and phylogenomic reconstructions and population structure analyses were inferred. Then, we used the combined data to generate species delimitation hypotheses. We subsequently determined the lineage diversity within each focal group implementing species delimitation methods. Additionally, time-calibrated species trees were inferred based on the results.
3. Landscape genomics: Landscape genomic analyses were performed on non-sympatric monophyletic lineages. We then identified shared barriers to gene flow in the system by merging all analyses.
4. Biogeographic history reconstruction: Time-calibrated species trees and phylogenomic reconstructions at the specimen level were employed to reconstruct the biogeographic history of each focal group.
5. Drivers of community assembly: We identified vicariant and dispersal events for each focal group and integrated them to assess the influence of mountain building and climate on the assembly of this reptile community.

### Taxon Sampling

To infer the number of focal groups in the Hajar Mountains, we assembled a dataset following the most updated reptile taxonomy (Uetz et al. 2023), including candidate species in the process of being described, representing a total of 284 squamate species and one outgroup (Supplementary Table S1). This dataset contained (i) all available species from the 8 genera with species endemic to the Hajar Mountains (i.e., not only the endemics to the Hajar Mountains), except for *Hemidactylus*, for which we only considered the “arid clade” (Carranza and Arnold 2006); (ii) at least one representative of each Squamata family; (iii) the only extant species of the order Rhynchocephalia (the tuatara, *Sphenodon punctatus*) as outgroup; and (iv) several key species to set 13 calibration points in the phylogenetic tree.

To investigate the genome-wide variation within each focal group, we selected specimens from each described endemic reptile species in the system, spanning throughout the whole mountain range, and including, when possible, individuals from all known genetic lineages of each species (Carranza and Arnold 2006; Carranza et al. 2016; de Pous et al. 2016; Tamar et al. 2016, 2019b, 2019a; Garcia-Porta et al. 2017; Simó-Riudalbas et al. 2017, 2018; Fattahi et al. 2020; Burriel-Carranza 2023a). We complemented each monospecific dataset with its closest described relative to root subsequent phylogenomic trees. We retrieved 53 ddRADseq samples of *Trachydactylus* from Burriel-Carranza et al. (2023b) and the rest were de novo produced in this study. The final dataset included 661 samples from 27 described species (19 endemic species and 8 outgroups). All samples were collected between 2006 and 2017, preserved in absolute ethanol, and stored at −20 °C until library preparation (see below “ddRADseq protocol”).

### Squamata Phylogenetic Reconstruction

To reconstruct a squamate time-calibrated phylogeny we included a total of 285 species (284 squamates and the tuatara). The dataset contained up to 15 genes retrieved from GenBank (Benson et al. 2012): 6 mitochondrial (*12S*; *16S*; Cytochrome oxidase subunit 1, *COI*; Cytochrome b, *cytb*; and NADH dehydrogenases 2 and 4, *ND2* and *ND4*) and 9 nuclear loci (the acetylcholinergic receptor M4, *ACM4*; the Brain Derived Neurotrophic Factor, *BDNF*; the oocyte maturation factor MOS, *CMOS;* the melanocortin 1 receptor, *MC1R*; neurotrophin-3, *NT3*; phosducin, *PDC*; the RNA fingerprotein 35, *R35;* and the recombination activating genes 1 and 2, *RAG1* and *RAG2;* see Supplementary Table S1 for further specifications and accession numbers).

Initial analyses using species tree approaches with *BEAST2 v.0.15.13 (Ogilvie et al. 2017) revealed challenges stemming from the high number of species in the dataset and the significant high levels of missing data in some markers (Supplementary Table S1). Convergence and stationarity were only marginally achieved after more than one billion generations and an extensive computation period exceeding 2 months. Additionally, stationarity was not achieved in some gene tree priors and results were not entirely congruent with previously available squamate phylogenies (Tonini et al. 2016; Šmíd et al. 2021; Tejero-Cicuéndez et al. 2022; see Dryad repository https://doi.org/10.5061/dryad.r7sqv9sj3). Several studies using simulation (Tonini et al. 2015) as well as empirical data (Pyron et al. 2014; Thompson et al. 2014) have reported that concatenation approaches can sometimes perform as well or better than species tree methods (Tonini et al. 2015). This is especially the case when levels of incomplete lineage sorting are low, few loci are used, or gene trees have weak phylogenetic signal (Bayzid and Warnow 2013; Tonini et al. 2015; Mirarab et al. 2016), conditions that our dataset and many other large-scale phylogenies generated with nuclear and mitochondrial markers obtained from traditional Sanger sequencing meet. Additionally, concatenation approaches seem to be more resilient to missing data than species tree approaches (Hovmöller et al. 2013; Jiang et al. 2014), making them particularly useful for large datasets generated from public repositories such as GenBank, where a complete representation of all selected markers might be impossible to obtain for the entire dataset (again, such as in our case). Therefore, we implemented a supermatrix approach, concatenating all genetic markers for the 285 samples.

A total of 1804 sequences were aligned using 2 different protocols for ribosomal and coding genes respectively. Mitochondrial non-transcribed *12S* and *16S* ribosomal genes were aligned by performing multiple sequence alignments using MUSCLE implemented in Geneious Prime 2020.2.5. Poorly aligned positions were eliminated with G-blocks (Castresana 2000) using low stringency options (Talavera and Castresana 2007). Coding mitochondrial (mtDNA) and nuclear (nDNA) DNA sequences were first trimmed up to the first codon position, then translated into amino acids, aligned with MUSCLE, and back-translated to nucleotides using TranslatorX (Abascal et al. 2010). Poorly aligned positions were also trimmed with G-blocks with low stringency options. Finally, all coding and ribosomal genes were concatenated in a single file using Geneious Prime 2020.2.5. Best-fit partitioning schemes and models of evolution were inferred using the software PartitionFinder v.2.1.1 (Lanfear et al. 2017), with each gene partitioned separately. The concatenated alignment had a total length of 10 713 base pairs (bp).

Phylogenetic relationships and divergence times were estimated with Bayesian Inference (BI) using BEAST2 v.2.6.4 (Bouckaert et al. 2019). We calibrated the phylogeny with 13 calibration points (Supplementary Table S2), avoiding fixing nodes that were of potential biogeographic interest for our analyses (i.e., no calibration points were placed within any genus that contained Hajar Mountain’s representatives). We constrained higher-level clades to match recent and supported squamate topology (Streicher and Wiens 2017; Šmíd et al. 2021; Tejero-Cicuéndez et al. 2022). The dataset contained 10 different partition schemes obtained from Partition Finder, and a discretized Gamma distribution with 4 categories was set for all partitions while independent substitution models were individually selected for each of them (Supplementary Table S3). We conducted 4 individual runs of 5 × 10^7^ generations, sampling every 10 000 generations with a Yule process tree prior. Convergence between runs and stationarity of each chain was checked with Tracer v.1.7 (Rambaut et al. 2018). Posterior distributions were combined with LogCombiner v.2.6.3 (Bouckaert et al. 2019), discarding 40% of the posterior trees as burnin and the maximum clade credibility tree was obtained calculating median heights in TreeAnnotator v.2.6.4 (Bouckaert et al. 2019).

### Identification of Focal Groups

We conducted an ancestral state reconstruction of mountain occupancy to infer the number of focal groups in this system. Focal groups were defined as all taxa stemming from the same colonization event. To determine the number of first colonization events and the age of colonization, we used the function “make.simmap” within the R package ‘phytools’ (Revell 2012) in the squamate multilocus phylogenetic tree. We assigned the state *Hajars*/*No Hajars* to each tip based on current species distribution, selected the most likely model under AIC criteria (all-rates-different; ARD) and ran 1000 simulations. This procedure also allowed us to differentiate between first colonization followed by within-mountain speciation, and independent colonization events within each mountain genus. Colonization events were considered when a node was present in the Hajar Mountains (more than 50% probability of its state as *Hajars*), and its parental node was recovered as *No Hajars*. We incorporated uncertainty to the age of colonization by considering a time range defined as the branch length between the colonization node and its parental *No Hajars* node, plus the 95% highest posterior density (HPD) interval on both nodes. We trimmed the ancestral state reconstruction results to the deepest node for each mountain genus with the exception of *Hemidactylus,* for which we only considered the ‘arid clade’, and *Omanosaura,* for which we added members of 2 closely related Eremiadini genera (*Acanthodactylus* and *Mesalina)*, as the whole genus is endemic to the mountain range.

### ddRADseq Protocol, Sequencing, Data Processing, and Dataset Building

We generated double digest restriction site-associated DNA (ddRAD) libraries with the protocol implemented in Peterson et al. (2012) using a combination of rare and common restriction enzymes (Sbf1 and Msp1, respectively) to digest 500 ng of genomic DNA and sequencing 75 base pairs (bp) single-end reads for a total of 661 individuals (Supplementary Table S4). Illumina raw reads were processed using iPyRAD v. 0.9.78 (Eaton and Overcast 2020). We filtered out low-quality sites (Phred score < 33), reads (≥3 missing sites) and sequences (<10 reads, >3 undetermined or heterozygous sites, or >2 haplotypes). Filtered reads were clustered and aligned using an 89% within- and between-sample clustering thresholds. The minimum number of samples per locus was left by default (>4) to retrieve the maximum number of loci possible for post-processing filtering. We further filtered our SNP data using an iterative filtering in an R script provided in Burriel-Carranza et al. (2023a). As suggested by O’Leary et al. (2018), we started with low cut-off values for missing data both by locus and by individual, and iteratively and alternately increased them to stricter values, discarding samples and genotypes not passing the filters. This approach let us identify and remove individuals and loci with low-quality values while overall recovering more high-quality loci than when only a hard filtering was applied (O’Leary et al. 2018). Filtering values for missing data allowance ranged from 98% to 60% of missing genotype call rate and missing data per individual, decreasing 2% between iterations. Then, different hard filters of missing genotypes were applied to further filter the dataset without removing individuals (Supplementary Table S5). Afterwards, non-biallelic SNPs were removed, we applied a minor allele frequency (maf < 0.05) filter and removed monomorphic sites.

All ddRADseq analyses were performed using one of the following dataset types. For Maximum Likelihood (ML) and Bayesian Inference (BI) reconstructions, we used concatenated loci files (c_loci) generated with iPyRAD after removing all individuals that did not pass the previously explained filters and retaining only loci that were at 60% of all individuals (Supplementary Table S5). For population structure, species delimitation, effective migration surface and species trees reconstruction we generated datasets of unlinked SNPs (one SNP per locus, uSNPs). However, for the genera *Asaccus* and the species *Pristurus rupestris* concatenated SNP (c_SNPs) datasets were used when building species trees, as we did not recover enough data to compute proper phylogenomic reconstructions with uSNPs.

### Lineage Discovery

We generated species delimitation hypotheses by following a 2-step workflow. We first reconstructed phylogenomic relationships within each focal group through ML and BI methods to determine well supported monophyletic groups. Then, we conducted ADMIXTURE v.1.3.0 (Alexander et al. 2009; Alexander and Lange 2011) analyses to evaluate a range of possible ancestral populations.

#### Phylogenomic reconstructions.

Using the c_loci alignments (Supplementary Table S5, datasets 25–34) for each focal group, we performed ML phylogenetic reconstructions inference with RAxML-ng v.1.0.2 (Kozlov et al. 2019), with a GTR + GAMMA model, a total of 100 starting trees (50 random and 50 parsimony) and 1000 bootstrap replicates. We also estimated BI with BEAST2 v.2.6.4 (Bouckaert et al. 2019). To reduce computational load, for each focal group we used 2 independent sets of 600 randomly picked loci present in at least 60% of samples (Supplementary Table S5, datasets 35–44). We calibrated each phylogeny by extracting the date of its deepest node from the squamate phylogenetic tree previously inferred and applying a normal distribution (see Supplementary Table S7 for specifications on calibration nodes). We selected a GTR model with 4 gamma categories, base frequencies were estimated, and a relaxed clock LogNormal was used. We conducted 2 individual runs of 10^8^ generations sampling every 10 000 generations. Convergence was checked with Tracer v.1.7 (Rambaut et al. 2018) and a burnin of 40% was applied. Convergence between sets of loci was achieved in all cases with the exception of *Ptyodactylus* and *Trachydactylus* for which we had to use all available loci (17,588 and 5115 loci, respectively) to achieve convergence between runs.

#### Admixture.

Using the uSNPs datasets for each described species (Supplementary Table S5; datasets 1–24), we inferred the population ancestry of each individual with ADMIXTURE v.1.3.0 (Alexander et al. 2009; Alexander and Lange 2011) using the script ‘admixture-wrapper’ (https://github.com/dportik/admixture-wrapper). We evaluated a range of possible ancestral populations from a minimum number of *K* = 1 to a maximum number of populations that ranged between *K* = 8–20 (depending on the maximum number of individuals in each group). For each K, we generated 15 replicates and 15-fold cross-validation to determine the most probable K.

Finally, we assembled different species delimitation models (SDM) by splitting each dataset into non-admixed, monophyletic deep lineages, to construct new SDMs with lumped taxa (see Supplementary Table S6 for specimen assignment to each model). All SDMs were assessed through Bayes Factor Delimitation (BFD* with genomic data; Leaché et al. 2014).

### Deep Lineage Delimitation and Time-Calibrated Species Trees

#### Species delimitation.

We used the ML and BI phylogenies together with the population ancestry from ADMIXTURE to design and evaluate up to 49 different configurations of species hypotheses with Bayes Factor Delimitation (BFD* with genomic data; Leaché et al. 2014; Supplementary Table S5, datasets 46–57). BFD* combines genomic data and coalescent methods to compare candidate SDMs containing different number of species. For each SDM we first estimated a species tree and then conducted a path sampling algorithm to calculate marginal likelihoods. Each marginal likelihood was then ranked and Bayes factors were used to determine the best species delimitation model for each dataset.

Species trees for each SDM were inferred with SNAPP v.1.5.2 (Bryant et al. 2012). This program uses a Bayesian multispecies coalescent framework to generate a species tree directly from biallelic markers (SNPs or AFLPs) without estimating gene trees. To avoid model violations (SNAPP assumes no gene flow) we only included non-admixed individuals. Moreover, since SNAPP is computationally intensive, we downsampled our datasets selecting 1–3 individuals with the highest coverage for each deep lineage (Supplementary Table S5, datasets 46–57). We also included the closest sister species of each described species to test for a single species hypothesis (see Supplementary Table S4 for specifications on which specimens were used). For all SNAPP analyses, mutation rates (*u* & *v*) were fixed to 1. The Yule prior (*λ = α × β*) representing the speciation rate was set to a gamma distribution, *α* was set to 2 and *β* was estimated by calculating the expected tree height on the c_loci datasets (maximum observed divergence between any pair of taxa divided by 2; extracted from datasets 25–34). We then used ‘pyule’ (https://github.com/joaks1/pyule) to determine the mean value of *λ* and calculated *β* accordingly. Theta prior (*θ*) was also set to a gamma distribution and the mean value of *θ* was estimated by averaging all genetic distances within each population. Each BFD* analysis was also conducted with default *λ* and *θ* priors and the most conservative result was kept (see Supplementary Table S6). Path sampling analyses were run for 20 steps with the following parameters: 500,000 MCMC generations sampling every 1,000, with an alpha of 0.3, 10% burnin and a preburnin of 50,000. Stationarity of all runs was checked and each step was run until ESS >= 200. Both SNAPP and BFD* were implemented in BEAST2 v.2.6.4 (Bouckaert et al. 2019).

#### Time-calibrated species trees.

After identifying the best SDM for each dataset, we reconstructed a time-calibrated species tree with SNAPP calibrating the deepest node in the phylogeny as suggested by Stange et al. (2018). We used the ‘snapp_prep.rb’ ruby script from (https://github.com/mmatschiner/tutorials) to prepare the SNAPP input file. Calibration dates (means and standard deviations) were extracted from the Squamata phylogenetic reconstruction (Supplementary Table S7) and were set to a normal distribution. We ran 3 independent runs of 3,000,000 generations, sampling every 50 generations. Convergence between runs and stationarity was checked with Tracer v.1.7 (Rambaut et al. 2018). Posterior distributions were combined with LogCombiner v.2.6.3, discarding 50% of the posterior trees as burnin and a maximum clade credibility tree was obtained calculating median heights in TreeAnnotator v.2.6.3 (both programs implemented within BEAST2 v.2.6.4; Bouckaert et al. 2019).

### Estimation of Genetic Connectivity and Barriers to Gene Flow

We implemented Fast and Flexible Estimation of Effective Migration Surfaces (FEEMS; Marcus et al. 2021) to determine connectivity corridors and barriers to gene flow. We generated a total of 14 datasets of uSNPs composed by non-sympatric monophyletic lineages (Supplementary Table S5, datasets 58–71) and a grid spacing ≈ 10 km between centroids. All non-gecko groups were excluded from the analysis due to low spatial coverage. We constructed a discrete global grid system with the R package ‘dggridR’ (Barnes et al. 2017) with a spacing resolution of 9 (≈ 10 km spacing between grid centroids) and clipped it with a self-generated perimeter of the mountain range (when samples spanned throughout the whole mountain range), or with a block-specific range (when specimens were only present in 1 or 2 mountain blocks). FEEMS uses a smoothing regularization parameter *λ* to tune each edge weight on the graph. Large values of *λ* (e.g., *λ* = 100) promote FEEMS to fit a model with most edges nearing the mean value, while small values of *λ* (e.g., *λ *= 0.00001) promote overfitting of the data (Marcus et al. 2021). To select the proper *λ* for each dataset, we selected the lowest, leave-one-out cross-validation value out of a range of lambdas between 1e^−6^ and 100 (Supplementary Table S5). With a custom R script (see Dryad repository https://doi.org/10.5061/dryad.r7sqv9sj3), we merged all 14 analyses into a single file to identify shared patterns across the entire range of the Hajar Mountains to make apparent the common geographic barriers to geneflow across species. Since the average migration weight equals 0 in all analyses, we were able to average all overlapping edges between the 14 analyses without having to apply any data transformation. We finally interpolated the results into a 1 km^2^ resolution raster for better visualization.

### Within-Mountain Biogeographic Reconstructions

We used the R package BioGeoBEARS (Matzke 2018) to reconstruct ancestral ranges for each focal group using the SNAPP time-calibrated species trees (see above). We divided the mountain range into 3 blocks (West, Central, and East), separated by the Wadi Jizzi gap and the Semail gap respectively (Fig. 1; see Garcia-Porta et al. 2017 for a similar delimitation of the mountain range), and assigned each tip to its respective mountain block. We considered that a lineage belonged to a geographic mountain block when the majority of its specimens were contained within the block. Only 2 taxa (*Omanosaura jayakari* lineage 2 and *Omanosaura cyanura* lineage 2) had to be assigned to 2 blocks simultaneously. We also added the initial states *Iran* and *Masirah* to account for out-of-the-mountain clades in the genera *Asaccus* and *Trachydactylus*, respectively. We set the maximum number of areas per node to 2 and performed ancestral reconstructions using the following models in BioGeoBEARS: Dispersal-Extinction-Cladogenesis (DEC; Ree and Smith 2008), DIVALIKE (Ronquist 1997) and BAYAREA (Landis et al. 2013). Best models were selected according to Akaike information criterion, correcting for small sample size (AICc; Akaike et al. 1973; Supplementary Table S8).

We followed the procedure implemented by Tejero-Cicuéndez et al. (2022) to track the incidence of mountain block colonization and vicariant events between blocks for each biogeographic reconstruction. We considered a block colonization event when a parental node was not present in a certain mountain block and at least one of its descendant nodes were. A vicariant event was identified when a parental node was widespread through 2 or more mountain blocks and each of its descendant nodes were in different blocks. To account for temporal uncertainty in the occurrence of these events, we included the branch length plus the 95% highest posterior density (HPD) interval of the age of both parental and descendent nodes in mountain block colonization events, and only the 95% HPD interval of the node age in which we recovered a vicariant event.

In addition, we implemented a model adequacy test by generating 1000 simulated biogeographic histories to quantify the extent to which the observed biogeographic history deviates from that expected from the best-fit model and the inferred phylogenetic history alone (Tejero-Cicuéndez et al. 2022). Then, we compared the number of observed events with the expected under the inferred model for all biogeographic events together, all vicariance events together, and each event separately, with the goal of identifying specific time intervals where the observed biogeographic history significantly deviates from model expectations. All biogeographic analyses were conducted within the R environment (R Core Team 2021) using the packages ggtree (Yu 2020), treeio (Wang et al. 2020) and packages within tidyverse (Wickham et al. 2019).

We also explored the biogeographic patterns and ancestral elevations at the specimen level (i.e., each tip represents a specimen in the phylogenetic tree) by conducting ancestral state reconstructions to each BI phylogenomic reconstruction (Supplementary Table S5, datasets 35–44) with the function “make.simmap” within the R package ‘phytools’ (Revell 2012). Phylogeographic traits were established according to the 3 discrete topographic discontinuities of the Hajar Mountains described above, and elevation traits were discretized into 3 categories: *lowland* (< 300 m)*, montane* (300–1500 m), and *high mountain* (>1500 m). These 3 elevation categories were chosen according to the climatic variability in the mountain range. *Lowland* areas were defined as all land below 300 m, and we used the climatic clusters defined in Carranza et al. (2018) to represent *montane* regions (clusters 10, 11, and 13–15) and *high mountain* areas (clusters 1–9 and 12). Subsequently, we selected the most likely model (ER, SYM or ARD) under AIC criteria (Supplementary Table S8) and ran 1000 simulations. Current elevation categories were compared to their most recent common ancestor’s state. If they differed, we considered it indicative of a “bottom-up” scenario when the ancestral node presented a lower state, or a “top-down” scenario when the ancestral node presented a higher state.

### Paleoclimate

We further investigated the dependence of all biogeographic events together, and vicariance events only, on global mean surface temperature estimates (Hansen et al. 2013) for the last 24 and 21 my respectively (ages are defined by the first biogeographic event and vicariant event recovered in our data). We fitted a GLS model over 10,000 permutations using a permutation-based method within the R package “RRPP” v. 0.4.0. (Collyer and Adams 2019). To account for temporal autocorrelation in the GLS, we built matrices of covariance incorporating the decay of temporal autocorrelation from an autoregressive model (AR1) within the R package “nlme” v. 3.1-162 (Pinheiro et al. 2023).

## RESULTS

### Squamata Phylogeny

**Figure 3.**
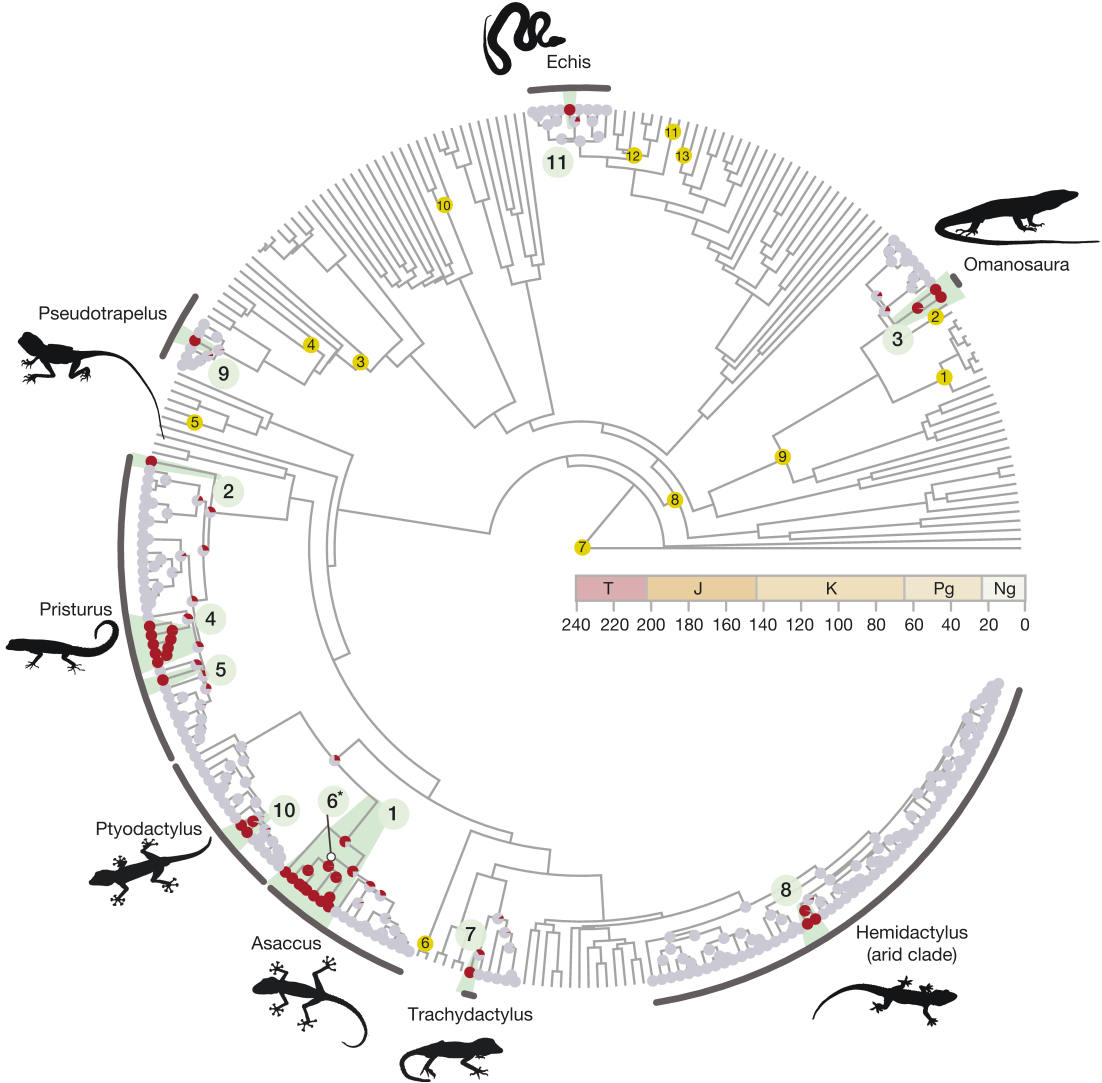
Multilocus squamate phylogeny of 285 species containing representatives from each family of the order Squamata and rooted with *Sphenodon punctatus* (the tuatara). The tree includes all available species from the genera present in the mountain range with the exception of the genus *Hemidactylus*, for which only the ‘arid clade’ was used. Nodes represent an ancestral state reconstruction of mountain occupancy in the squamate phylogenetic tree. Gray nodes refer to the state “*No Hajars*” and red nodes refer to the state “*Hajars*.” Green numbered circles show the 11 independent colonization events ordered by colonization age. Colonization event 6 was only recovered in the biogeographic reconstructions with genomic data but is shown here for visualization purposes. The genera present in the mountain range are shown highlighted in black. In yellow the 13 calibration nodes used for the phylogenetic reconstruction (Supplementary Table S2). T: Triassic; J: Jurassic; K: Cretaceous; Pg; Paleogene; Ng: Neogene. For a more detailed tree including species names and ages of colonization see Supplementary Fig. S1. A color version of this figure appears in the online version of this article.

Multilocus squamate phylogenetic reconstruction (Fig. 3) yielded a similar topology and crown age estimates of the clades of interest as in previously published phylogenies (Šmíd et al. 2021; Tejero-Cicuéndez et al. 2022). More importantly, including all species in the same phylogenetic framework allowed us to compare node ages between all Hajar Mountain’s endemics, a key aspect to answering our integrated biogeographic questions and calibrate the Bayesian Inference trees and species trees generated with ddRADseq. For a phylogenetic tree with species names and further information see Supplementary Fig. S1.

### ddRADseq Dataset Assembly

We obtained a total of 15.84 × 10^8^ raw reads with 15.80 × 10^8^ passing the quality filters. Throughout the post-processing filtering, we identified and discarded 85 individuals due to low sample coverage. This resulted in an increase in the average reads per sample from 2.4 × 10^6^ to 2.7 × 10^6^. Reads retained per sample after sample trimming ranged between 1.9 × 10^5^ and 10.5 × 10^6^ with a median of 2.4 × 10^6^ reads (Supplementary Table S4). Datasets generated for lineage discovery, population structure, and lineage delimitation analyses, presented 13.14% missing data on average, the number of loci ranged between 18,982 and 491, and contained between 173 and 7 samples. For detailed summary statistics on each dataset, refer to Supplementary Table S5.

### Lineage Discovery, Deep Lineage Delimitation, and Phylogenetic Discordances

We generated primary species hypotheses through ML and BI phylogenies, and ADMIXTURE. We evaluated each primary hypothesis with BFD* and generated time-calibrated species trees with SNAPP to perform the biogeographic assessments.

#### Phylogenomic reconstructions.

Maximum likelihood (ML; Supplementary Figs. S2–S11) and Bayesian inference phylogenies (BI; Supplementary Figs. S12–S21) yielded similar topologies in all datasets and posterior support (pp > 0.95) or bootstrap support (bp > 85) was generally recovered in all major clades. The phylogenomic reconstruction of *Trachydactylus hajarensis* is the only case showing discordances between analyses, possibly due to historic gene flow between populations (Burriel-Carranza et al. 2023a).

We obtained, for the first time, robust support for the position of *Asaccus margaritae* within the *Asaccus* genus as sister to the clade conformed by *A. platyrhynchus*, *A. arnoldi* and *A. gallagheri* (Supplementary Figs. S2 and S12). We also found strong support for 2 Iranian *Asaccus* clades instead of the previously reported monophyly of *A. nasrullahi*, *A. griseonotus* and *A. elisae* (Carranza et al. 2016; Simó-Riudalbas et al. 2018; Fattahi et al. 2020).

#### ADMIXTURE.

The minima of the cross-validation error varied substantially between groups (Supplementary Table S5). *Asaccus arnoldi*, *A. caudivolvulus*, *A. gardneri*, and *Pseudotrapelus jensvindumi* most probable *K* scenario was *K* = 1. In the other species, we recovered between 2 and 5 populations per species, except for *Pristurus rupestris*, for which the best cross-validation values ranged between *K* = 11 and *K* = 20. In that case, we conducted subsequent ADMIXTURE analyses within *P. rupestris* subclades to better delimit its structure. Overall, we recovered between 42 and 56 population groups as distinct species hypotheses (Supplementary Figs. S21–S25 for best K scenario; Supplementary Figs. S26–S46 to other ADMIXTURE configurations).

#### BFD*.

Bayes Factor Delimitation* showed almost no difference between using default parameters and species-specific priors. When different, we kept the best BFD* result from the analysis with the most conservative species hypothesis, being the one more similar to the current described diversity (Supplementary Table S6). We evaluated 49 different species hypotheses ranging from 19 (number of described species) to 55 putative species. BFD* supported up to 49 independent evolutionary units, with the highest diversity being within the genus *Asaccus* (13 identified lineages) followed by *Pristurus rupestris* (12 lineages), *Omanosaura* and *Trachydactylus hajarensis* (4 lineages each), *Echis*, *Hemidactylus*, *Pristurus celerrimus* and *Ptyodactylus* (3 lineages each), and *Pristurus gallagheri* and *Pseudotrapelus jensvindumi* (2 lineages each).

#### Species trees.

Time-calibrated species trees were concordant with previously published phylogenies and the main ML and BI clades. We obtained the same *Asaccus* topology as in the ML and BI phylogenies. Dates of first within-mountain diversification ranged from 13.7 to 0.12 ma between groups (Supplementary Figs. S47–S54).

### Genetic Connectivity and Barriers to Gene Flow

**Figure 4.**
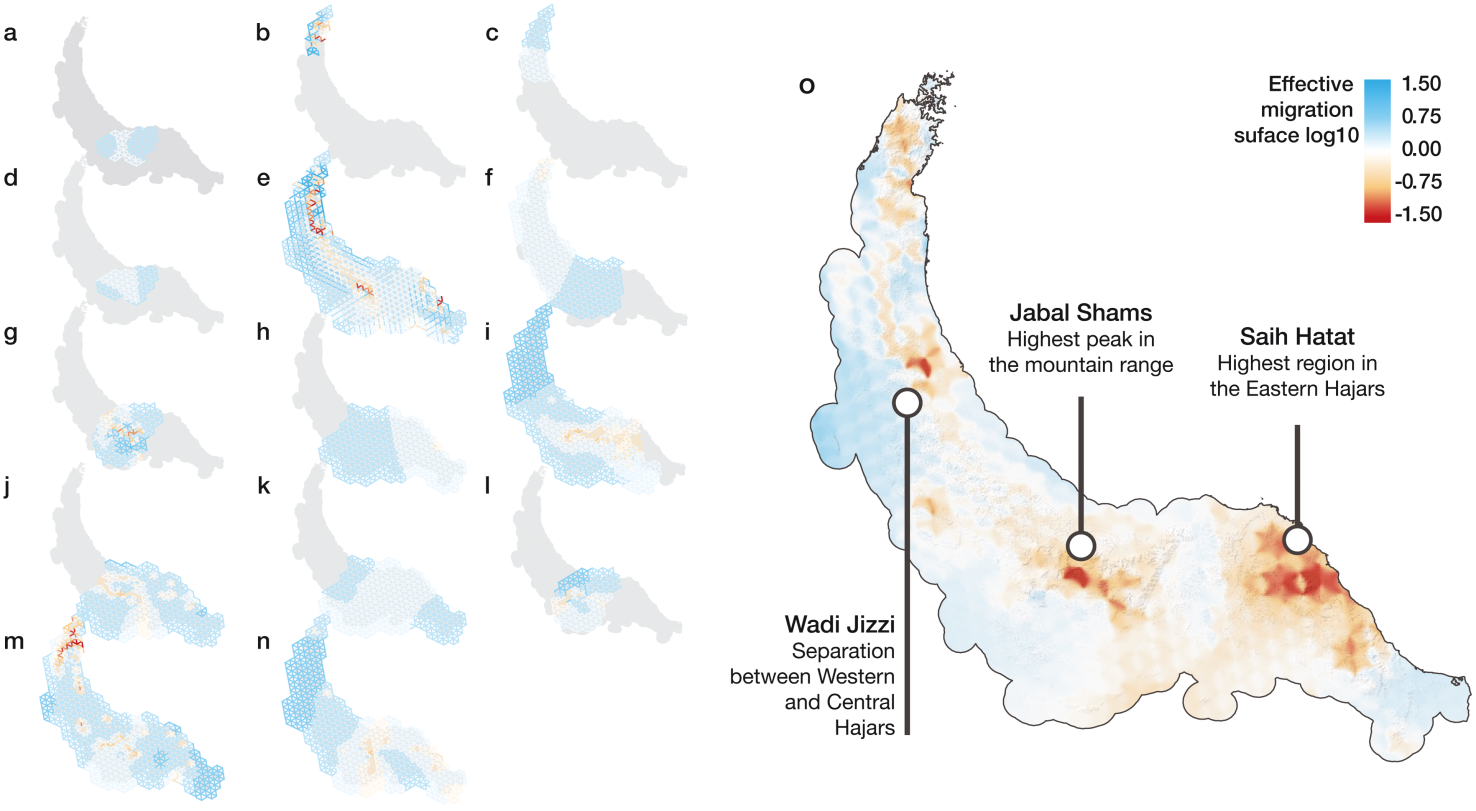
Barriers to gene flow calculated with Fast Estimation of Effective Migration surfaces (FEEMS) analyses. Blue colors represent higher than average effective migration, red colors are lower than average effective migration (genetic barriers); Captions a-n represent the fit of FEEMS with the best cross-validation smoothing regularization parameter (*λ*) for the following datasets: a) *Asaccus montanus*, *λ* = 100; b) *A. gardneri*, *λ* = 0.0023; c) *A. margaritae*, *λ* = 100; d) *A. platyrhynchus*, *λ* = 100; e) *A. arnoldi* and *A. gallagheri*, *λ* = 1.8 × 10-5; f) *Pristurus celerrimus*, *λ* = 14.38; g) *P. gallagheri, λ* = 0.006; h) *P. rupestris* lineages 5 and 6*, λ* = 100; i) *P. rupestris* lineages 7–11*, λ = 0.78*; j) *P. rupestris* lineages 1-3*, λ* = 0.78; k) *Hemidactylus hajarensis, λ* = 37.62; l) *H. luqueorum, λ *= 0.042; m) *Ptyodactylus orlovi* and *P. ruusaljibalicus, λ* = 0.042; n) *Trachydactylus hajarensis*, *λ* = 0.11. o) Averaged result of all 14 analyses of FEEMS interpolated to a raster of 1 km resolution. The hillshade in the background allows to locate the main topographic features of the mountain range. White circles describe the topographic features in the regions of greater genetic barriers. A color version of this figure appears in the online version of this article.

*Trachydactylus hajarensis*, *Ptyodactylus*, *Pristurus gallagheri,* and *Pristurus rupestris* lineages 1–3 and 7–11 were the groups with the greatest values of genetic resistance (Fig. 4a–n). When all 14 analyses were merged together (Fig 4o), 3 main genetic barriers were apparent across the mountain range. Two of them coincided with the Jabal al Akhdar and the Saih Hatat culminations, the 2 highest regions of the Hajar Mountains (Fig. 1). The third was found about 25 km northwest of the Wadi Jizzi Gap, one of the major drainage systems in the mountain range, which has been previously used to delimit the end of the Central Hajars and the start of the Western Hajars (Garcia-Porta et al. 2017). Overall, high and complex topographies were recovered as areas with less migration than average.

### Reptile Biogeography Through the Orogeny of the Hajar Mountains

#### First colonizations.

First colonization events were estimated with an ancestral state reconstruction of presence in the mountains conducted on the multi-locus squamate tree. We found 10 independent colonization events with a single colonization event in each genus, except in the genus *Pristurus,* where we retrieved 3 independent colonization events (Fig. 3). The only case of extirpation from the mountain range was found in the genus *Asaccus*, where the Iranian clade diverged from the Arabian *Asaccus montanus* between 28.4 and 19.3 ma. Colonization dates range between 70.39 ma and 0.1 ma with 9 out of 10 events having occurred after or during the main uplift of the Hajar Mountains in the late Eocene (40–30 ma). The colonization event of *Asaccus* is the only case that may have preceded the main uplift event. However, temporal uncertainty in *Asaccus* is very high, ranging between 70.39 and 28.05 ma (Fig. 3, Supplementary Fig. S1), also including the main uplift temporal range.

#### Intra-Mountains approach:

*Biogeographical assessment.* —The biogeographic histories for each clade at the lineage and specimen levels are summarized in Supplementary Figs. S12–S21, S47–S54. The topology obtained through phylogenomic reconstructions of the genus *Asaccus* resulted in a different biogeographic history than the inferred with the squamate multilocus phylogeny. In this case, we did not recover an extirpation from the Hajar Mountains, but 2 independent colonization events, increasing the number of independent colonization events from 10 to 11 (Supplementary Figs. S12 and S47). Although previous BI and species tree reconstructions with Sanger data had yielded the same topology as in our squamate phylogenetic reconstruction (Carranza et al. 2016; Tamar et al. 2019), none of those strongly supported the monophyly of the Iranian *Asaccus* or the position of *A. margaritae* as sister to *A. gardneri* and *A. caudivolvulus*. Contrastingly, the species tree reconstructed with genome-wide SNPs has a posterior probability of 1 in all nodes and, together with the significantly higher number of SNPs retrieved with ddRADseq, we find this to be a more reliable scenario than the one obtained through the multi-locus phylogeny. The ancestral range reconstruction at the specimen level (Supplementary Figs. S12–S21) enabled us to reconstruct the biogeographic history of *Pseudotrapelus jensvindumi* and *Pristurus gallagheri*, which could not be achieved with BioGeoBEARS due to having less than 3 lineages each. Cases where many individuals are in a different block than that of their identified lineage are scarce and mostly occur in contact regions between clades (e.g., *Pristurus rupestris* lineages 5 and 6) or in groups with high dispersal capabilities (e.g., *Omanosaura cyanura* and *Pseudotrapelus jensvindumi*).

**Figure 5.**
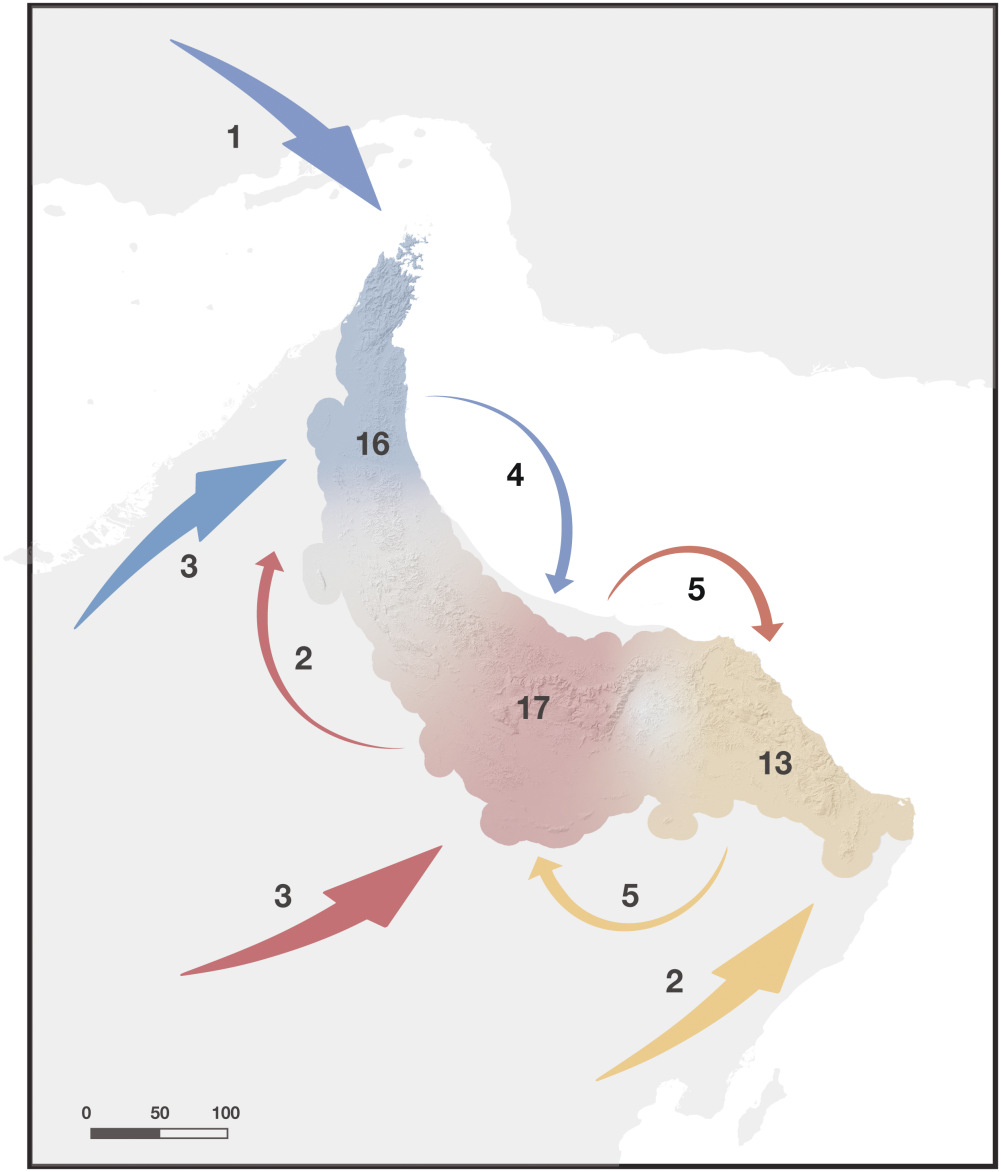
Map representing first colonization events (large arrows arriving to the mountain range), number of first colonization events at each mountain block (shown by numbers next to first colonization arrows), dispersion events between mountain blocks (represented by smaller arrows and numbers from one block to another), and current endemic block diversity (numbers within the mountain range) in the Hajar Mountains. Blue: Western Hajars; Red: Central Hajars; Yellow: Eastern Hajars. A color version of this figure appears in the online version of this article.

**Figure 6.**
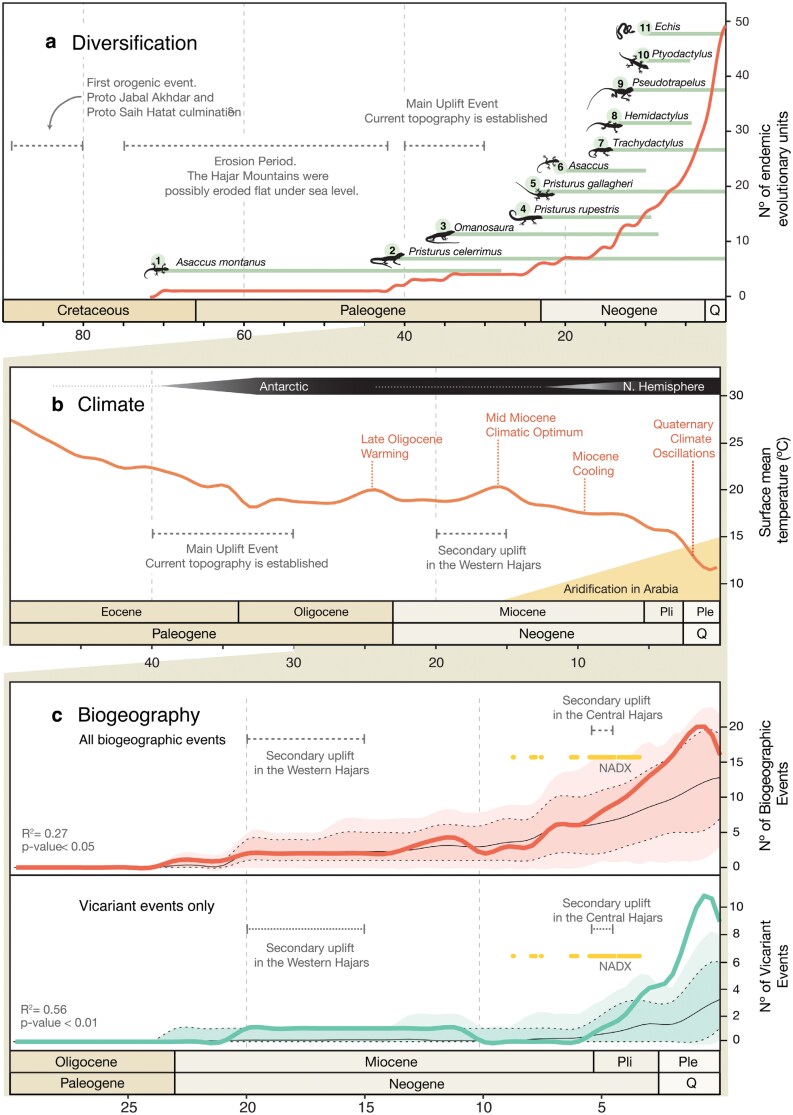
Sequence of events for a) diversification, b) climate and c) biogeography in the Hajar Mountains of southeastern Arabia. a) cumulative number of endemic reptile deep lineages (obtained through Bayes Factor Delimitation*; *with genomic data) through time. Horizontal bars represent the time range of first colonization events defined as the branch length between the first colonization node and its parental *No Hajars* node plus the 95% highest posterior density (HPD) interval of both nodes in the squamate tree reconstruction (Fig. 3), with the exception of colonization event 6, for which we extracted the branch length from the genomic species tree reconstruction (Supplementary Fig. S12). b) Climatic sequence of events including global surface mean temperature estimates (red line; modified from Zachos et al. 2001, 2008; Hansen et al. 2013); Established ice sheets of the Antarctic and of the Northern hemisphere are shown with white to black bars on the top. The onset of North Africa and Arabia desertification since the Mid Miocene climatic optimum is shown bellow. c) Observed (colored lines) and expected (background coloration and black lines) incidence of biogeographic events through time. Colored background represents 1000 random biogeographic histories simulated under the best-fit model with BioGeoBEARS (DIVALIKE in all cases). Black dashed-lines enclose the 95% confidence interval of the simulations and thin black lines represent the average. In red (top) all biogeographic events (vicariant and dispersal events) are represented together. In green (bottom), vicariant events only. Observed vicariant events deviate from that expected from the best-fit model and the inferred phylogenetic history around 3.5 ma, suggesting that external forces were promoting vicariance. We found a significant relationship between mean global temperatures and both biogeographic event reconstructions (**c**, bottom-left corner in both subpanels), even when accounting for temporal autocorrelation; Yellow bars represent Neogene hyper-arid periods in the Arabian Peninsula (extracted from Böhme et al. 2021); NADX: Neogene Arabian Desert Climax. Gray horizontal dashed-lines show the orogenic history of the Hajar Mountains through captions a,b and c. A color version of this figure appears in the online version of this article.

We conclusively identified the region of first entry into the mountain range of 9 out of the 11 independent colonization events (Fig. 5). Four clades colonized the Hajar Mountains through the Western block (the second *Asaccus* colonization event, *Echis omanensis, Pristurus celerrimus*, and *Ptyodactylus*), 3 clades entered through the Central Hajars (*Hemidactylus*, *Pseudotrapelus jensvindumi* and *Pristurus gallagheri*), and 2 clades entered through the Eastern Hajars (*Pristurus rupestris* and *Trachydactylus hajarensis*). Across all clades, biogeographic reconstructions with BioGeoBEARS yielded 15 within-mountain block colonization events (3, 5, and 5 in the Western, Central, and Eastern blocks, respectively), 15 vicariance events between the blocks, and one vicariance event between Iran and the Western block. First biogeographic events started accumulating around 23 ma, but the overall biogeographic incidence is scarce until 5 ma when most of the events occurred (Fig. 6).

When comparing the observed incidence of all biogeographic events through time with the model expectations (which reflect the dynamics of species accumulation alone), we did not find any clear deviances (Fig. 6c). This means that the reconstructed biogeographic history falls within the random distribution generated with the 1000 simulated biogeographic histories and that a large part of the observed reconstruction might be explained by the shape of the phylogeny, the number of lineages through time, and the best-fit biogeographic model (DIVALIKE in all datasets).

However, when looking into each type of event separately (Supplementary Fig. S55), we found that this model does not fully capture the temporal dynamics of each biogeographic event. We found that the incidence of vicariance events was higher than expected during the last 3 my (Fig. 6c), especially for Central-East and Central-West vicariance events (Supplementary Fig. S55). Furthermore, we recovered that the split between the Iranian and Western Hajars’ *Asaccus* clades was slightly different than expected, coinciding with a secondary uplift of the Hajar Mountains’ Western block that further isolated both landmasses (Jacobs et al. 2015). The number of observed block colonization events does not significantly differ from the expected with the exception of the Central block colonization events, which are slightly higher than expected between 5 and 3 ma.

#### Intra-Mountains approach:

*Elevation assessment.* —Even though single- to few-specimen transitions within a lineage can represent contemporaneous metapopulation dispersal into different elevations, they could rather be reflecting the capacity of these individuals to roam through less-suitable areas, or even a lack of precision in the geographic coordinate collection of the sample. Since usage of discrete barriers cannot fully include the nuances of continuous species distributions, we opted for a cautious interpretation of our results. Hereby, we only report elevation changes at the lineage level considering the state of the majority of individuals within each identified clade (Supplementary Figs. S12–S21). Ancestral elevation reconstructions showed several cases of ‘bottom-up’ dispersal from lowland to montane linages, or from montane to high mountain lineages in *Asaccus platyrhynchus*, *A. gallagheri* lineage 3, *A.margaritae*, *Pristurus celerrimus* lineage 3, *Pristurus rupestris* lineages 4, 6, 10, and 12, and *Trachydactylus hajarensis* lineage 2. On the other hand, ‘top-down’ transitions were only observed in *Asaccus margaritae* lineage 3 and *Pristurus rupestris* lineage 2. Six of the 9 “bottom-up” scenarios occurred between clades inhabiting the Central Hajars and, out of those, 4 coincided with a Central Block colonization. This suggests that the colonization of the region with the highest topographic relief of the mountain system comes along with dispersal and adaptation to higher altitudes.

In the non-gecko species, we did not find signals of a whole lineage transition between elevation categories, probably due to their higher dispersal capabilities. Ultimately, the reconstructed state at the deepest node in each analysis revealed that *Ptyodactylus*, *Trachydactylus* and the endemic species within the second *Asaccus* colonization, most likely originated from lowland-dwelling ancestral populations that colonized the mountain range and posteriorly adapted to its higher topography.

### Paleoclimate

The biogeographic dynamics of the endemic reptile fauna of the Hajar Mountains are significantly dependent on the temperature shifts of the last 20 my (Fig. 6c). When accounting for temporal autocorrelation, there is still a significant relationship between temperature and biogeographic events. Overall, the proportion of variance explained by global mean surface temperatures is higher in vicariant events only (*R*^2^ = 0.56) than in all biogeographic events together (*R*^2^ = 0.27).

## DISCUSSION

In the present study, we have assembled an extensive and unprecedented genomic dataset for the entire endemic squamate diversity of a mountain range from one of the major arid areas in the world. A key strength of this work is the integration of different sequencing approaches which build upon each other while capitalizing on their respective strengths. On the one hand, Sanger sequencing has provided a robust, deep-time phylogenetic framework, allowing for the identification of focal groups and a broad understanding of how this endemic reptile community first set foot in the Hajar Mountains (Fig. 3). Conversely, each independent genomic dataset has allowed us to delve into the intricacies of each focal group, assessing their diversity, systematics, and evolutionary history in depth. Altogether, this has allowed us to study the squamate diversity of the Hajar Mountains at multiple scales of biodiversity organization, obtaining from new highly detailed insights (i.e., species boundaries) to an overarching understanding of biodiversity patterns in the region (i.e., general biogeographic patterns across the entire mountain range), broadening our understanding of the buildup of montane communities.

### Patterns of Reptile Mountain Diversity

Reptiles, as ectotherms, rely on the use of direct sunlight to regulate their body temperature (Porter and Gates 1969). It is not surprising then, that contrasting patterns of reptile diversity across the elevational gradient are observed between humid forested mountains and arid mountain ranges (McCain 2010). In the former, reptiles have limited opportunities to utilize radiant heat, resulting in reptile diversity being concentrated in the warmest temperatures within the lowest 300 m of these mountains (McCain 2010). In the latter, reptiles seem to benefit from the increased radiant heat surfaces provided by the lack of dense vegetation, the lower humidity, and less cloud cover, resulting into greater diversity along the elevation gradient (McCain 2010). Concordant with the patterns of reptile diversity in other arid mountains (McCain 2010), reptile diversity in the Hajars is widely distributed across the elevation gradient, with the peak of species richness being found between 500 and 1000 m above sea level (asl) and with up to 12 species reaching above 2000 m asl (Supplementary Figs. S12–S21; Burriel-Carranza et al. 2022).

The integration of all analyses of estimation of effective migration surfaces (Fig. 4) has provided a novel perspective to investigate the patterns of reptile diversity for an entire community. This revealed that the highest peaks of the mountain range (i.e., Jabal Shams in the Central Hajars and Saih Hatat in the Eastern Hajars; Fig. 1) are acting as geographic barriers to gene flow for the entire guild, while lower regions act as corridors to lowland dwellers (Fig. 4), which thus confirms previous findings for some eastern Arabian squamates (Simó-Riudalbas et al. 2019; Pola et al. 2024). Moreover, as seen in population structure results (Supplementary Figs. S22–S25) the majority of deep lineages are currently endemic to specific mountain blocks, even with several examples of highly divergent populations between north- and south-facing slopes within the same block (e.g., *Asaccus platyrhynchus*, *Pristurus rupestris* and *Pristurus gallagheri*; Supplementary Figs. S22 and S24). Therefore, it seems that both avoidance of low and high elevations is driving diversification. On the one side, avoidance of low elevations (i.e., Wadi Jizzi and Semail gaps) is driving species formation on deeper divergences, while on the other, avoidance of higher elevations is promoting genetic structure within species.

The phylogeographic patterns of reptile diversity in the Hajar Mountains show that this mountain range has had a dynamic role, acting at the same time as a cradle (an area of rapid speciation) and a museum (an area of long-term persistence of species) for reptile diversity. For instance, *Pristurus rupestris* as well as the species within the second colonization of the geckos from the genus *Asaccus* (i.e., clade containing all species in the Hajars except *A. montanus*) have undergone rapid speciation in the mountain range leading to almost half of the current reptile diversity of the Hajar Mountains (47% of all deep lineages). Morphologically cryptic taxa occupying similar habitats, and even with sympatric distributions, are commonly found in both groups, thus, suggesting a case of non-adaptive speciation, probably driven by the isolation of populations through range contractions, and leading to allopatric or parapatric speciation.

Conversely, the role of the Hajar Mountains as a reservoir of reptile diversity is exemplified by the presence of *Asaccus montanus* and *Pristurus celerrimus* in the mountain range. Both *A. montanus* and *P. celerrimus* represent deep phylogenetic lineages, sister to all other species within their respective genera (Fig. 3). This highlights the biogeographic importance of the Hajar Mountains as a possible origin for reptile biotas that have afterwards dispersed across the Afro-Arabian deserts (*Pristurus*) and the Irano-Anatolian mountains (*Asaccus*).

### Diversification in Arid Mountains

The interaction between climate and the topographic complexity in mountains produces highly intricate environmental heterogeneity, sometimes conferring dramatic changes in rainfall patterns and vegetation over small distances, and ultimately leading to high species diversity and endemicity in these systems (Perrigo et al. 2020). This also holds true for arid mountains which, despite their low pluviosity and relatively sparser vegetation (i.e., traditionally associated with low species richness), present high diversity and endemicity levels along the elevation gradient (McDonald et al. 2021; Kohli et al. 2022).

In the present study, species delimitation methods using genomic data under the multispecies coalescent have identified up to 49 independent evolutionary units across 8 genera from 6 Squamata families: Gekkonidae, Sphaerodactylidae, Phyllodactylidae, Lacertidae, Agamidae and Viperidae. This is a staggering amount of species-level diversity especially considering that the Hajar Mountains are a rather small mountain range. However, even though some lineages have diverged from their sister clades more than 10 ma, we must proceed with caution when suggesting species splits. There is a strong debate on whether species delimitation methods with genomic data actually delimit species or rather capture population splits (Sukumaran and Knowles 2017; Ree and Sanmartín 2018; Leaché et al. 2019, 2021; Stanton et al. 2019; Chambers and Hillis 2020; Bamberger et al. 2022; Burriel-Carranza et al. 2023a), since the amount of information available with genomic data tends to promote oversplitting. Therefore, further lines of evidence and in-depth taxonomic work should be taken into consideration to discern if the identified lineages could actually represent new species.

Nonetheless, our results show that there are still high levels of undescribed reptile diversity in this mountain range, which can likely be extended to global arid regions. Importantly, this study highlights the role of arid mountains in generating and preserving diversity, as well as the urgent need of thorough taxonomic assessments in montane fauna.

### Drivers of Mountain Reptile Diversity

The outstanding diversity of squamate reptiles in the Hajar Mountains originated from 11 independent colonization events, which have subsequently dispersed and radiated throughout the mountain range. Our phylogeographic reconstructions showed that most of the Hajars’ endemic diversity originated within the past 30 my, a time when the mountain range had most likely reached its present topography (Hansmann et al. 2017). Therefore, most colonization events seem to be the result of communities dispersing into an already formed montane environment. Moreover, the lack of deviance in biogeographic incidence from the simulations during uplift episodes suggests that periods of mountain building have not been a particularly significant driver for the biogeographic processes of its endemic communities (Fig. 6c), with the exception of the first colonization of *Asaccus*, which may constitute a case of passive uplifting (Fig. 6a). In a study examining the geological origin and uplift history of the Hajar Mountains, Hansmann et al. (2017) suggested that before its main uplift event 40 ma, the Hajar Mountains might have been completely eroded under sea level. However, the possible presence of *Asaccus montanus* prior to that time in the Central Hajars (the current highest region of the mountain range) suggests that not all the Hajar Mountains were below sea level. This species might represent a remnant of the ancestral residents of the proto-Hajar Mountains, which subsequently went through a process of passive uplifting of its populations when the mountain range rose during the Late Eocene. Concordantly, *A. montanus* represents the only known reptile species in Oman and the UAE that exclusively occurs above 1800 m.

Rather than the processes of mountain building, paleoclimatic dynamics seem to have driven the assembly of this montane community. The excess of vicariance events found from the Pliocene onwards (Fig. 6c) show a strong correlation with the desertification in North Africa and Arabia since the mid Miocene (Flower and Kennett 1994; Pokorny et al. 2015), even when accounting for temporal autocorrelation (Fig. 6c). Since the mid Miocene, an increasing number of hyper-aridity episodes in Arabia have been recorded. Among those, the Neogene Arabian Desert Climax (NADX; Böhme et al. 2021) constituted an extremely long period (from 5.9 to 3.3 ma) during which Arabia served as a vicariant agent for African and Eurasian mammals (Böhme et al. 2021). The observed incidence of vicariant events starts to deviate from the expected while overlapping with the NADX (Fig. 6c), thus suggesting that this 2.5 my hyper-arid period could have had a key role, fostering range contractions and population fragmentations, consequently leading to vicariant speciation (McDonald et al. 2021). However, the NADX alone cannot explain the entire deviation of observed vicariance events from model expectations, since most of the unaccounted vicariance occurred during the Quaternary (Fig. 6).

The climatic cyclicity of the Quaternary ice-ages has been largely proposed as a key driver of speciation for montane Palearctic communities (Kotwicki and al Sulaimani 2009; Rahbek et al. 2019b). The swift shifts between cold and temperate climates impelled rapid and repeated dynamics of range splitting and secondary contacts, promoting species diversification (Rahbek et al. 2019b). However, the effect of such periods on communities inhabiting lower, warmer latitudes are still not well understood. At a regional scale, periods of global high-latitude glaciations led to increased aridity in Arabia. Global sea level falls of up to 130 m entailed the depletion of the Arabian Gulf, which increased the northwestern Shamal winds from Iran, and promoted desert formation and hyper-arid conditions in the Arabian Peninsula (Glennie and Singhvi 2002). On the other hand, interglacials brought increased humidity to the Hajar Mountains with sometimes torrential and continuous rains provided by a more northerly latitudinal range of the southwest monsoon (Rodgers and Gunatilaka 2003). The unusual Bajada, an immense alluvial fan extending about 40,000 km^2^ southwest to the Hajar Mountains, provides a hint of the scope of such humid periods (Rodgers and Gunatilaka 2003).

Climatic fluctuations between hyper-arid and temperate conditions seem to have impelled recurrent population fragmentation and further speciation in isolation, leading to the high levels of vicariance events observed in the biogeographic analyses (Fig. 6c). Although the resolution of the biogeographic reconstructions does not allow for a precise investigation to determine the effects of arid and humid regimes in the evolutionary history of the Hajar Mountains’ fauna, we argue that hyper-arid conditions could have promoted population fragmentation, pushing species upwards to colder areas or separating populations into micro-refugia in isolated wadis with enough water supply. Periods of increased humidity would have then allowed for populations to rejoin, either merging back to a single unit, or driving character displacement and speciation. Altogether, this system shows that Quaternary glaciations not only have had a profound influence over the evolution of high-latitude fauna, but also influenced the patterns of reptile diversity in the arid regions of the Arabian Peninsula. Interestingly though, glacial and interglacial periods would have had opposite effects in the upslope and downslope dispersal of Palearctic and Arabian montane faunas (Rahbek et al. 2019b).

The factors influencing the buildup of reptile diversity in the Hajar Mountains align with other systems where climate has also played a key role in shaping the evolutionary history of their fauna (Smith et al. 2014; Renner 2016). This might particularly hold true for geologically old systems (such as the Hajar Mountains), where community replacement may have occurred due to bottlenecks during the onset of the ice-ages (Perrigo et al. 2020). However, while Pleistocene global ice ages seem to have influenced the endemic reptile fauna of the Hajar Mountains, it cannot solely explain the excess of vicariance events as these started accumulating prior to the ice ages. It appears then, that the ice ages may have intensified a pre-existing trend, promoted by the constant aridification since the mid Miocene that led to the formation of the Sahara and Arabian deserts.

## Conclusions

This study shows, for the first time, how a whole reptile community has originated, diversified and maintained in an arid mountain range, combining genomic and biogeographic perspectives. We offer new insights onto the buildup of arid mountain communities and to the main drivers influencing their biogeographic and evolutionary processes. Here, we observe that climatic oscillations and hyper-arid periods have had a crucial role in building this montane community, promoting vicariance levels that exceed those expected from the best-fit biogeographic models. We also revealed an extraordinary level of cryptic diversity, highlighting the urgent need for further taxonomic and evolutionary research in arid regions. As exemplified by the Hajar Mountains, global arid areas likely hold high levels of undescribed diversity, providing crucial insights into the shaping of the ecosystems by environmental changes over time, and how they may continue to evolve in the face of a rapidly changing climate.

Finally, this study not only serves as a benchmark for reptile communities or mountain biotas, but also presents a valuable methodological framework that can be applied to other biological systems. The integration of multiple sequencing approaches together with biogeographic analyses exemplify a novel, flexible, and scalable approach that can be applicable to any taxonomic level (e.g., mammals, butterflies, beetles, birds), in any discrete biogeographic region (e.g., a mountain range, a lake, an island, an archipelago), and with any type of environment (e.g., arid, temperate, tropical).

## SUPPLEMENTARY MATERIAL

Data available from the Dryad Digital Repository: https://dx.doi.org/10.5061/dryad.r7sqv9sj3.

## Data Availability

Supplementary material, including supplementary tables and figures, raw demultiplexed ddRADseq reads, datasets, and scripts used in the present work can be found in the Dryad repository at https://doi.org/10.5061/dryad.r7sqv9sj3.
